# Magnetic Resonance Imaging Feature Analysis and Evaluation of Tubal Patency under Convolutional Neural Network in the Diagnosis of Infertility

**DOI:** 10.1155/2021/5175072

**Published:** 2021-09-17

**Authors:** Na Liu, Qingling Ren

**Affiliations:** First Clinical Medical College, Nanjing University of Chinese Medicine, Nanjing 210023, Jiangsu, China

## Abstract

To explore the diagnostic value of MRI image features based on convolutional neural network for tubal unobstructed infertility, 30 infertile female patients were first selected as the research objects, who admitted to the hospital from May 2018 to January 2020. They all underwent routine MRI examinations and CNN-based MR-hysteron-salpingography (HSG) examinations, in order to discuss the diagnostic accuracy of the two examinations. In the research, it was necessary to observe the patients' imaging results, calculate the diagnosis rate of the two examination results, and analyze the application effect of the CNN algorithm, thereby selecting the best reconstruction method. In this study, the analysis was conducted on the basis of no statistical difference in the baseline data of the included patients. The results of undersampling reconstruction at 2-fold, 4-fold, and 6-fold showed that CNN for data consistency layer (CNN_DC) had a better effect, and its peak signal-to-noise ratio (PSNR) was lower sharply than that of the other two reconstruction methods, while the normalized mean square error (NMSE) and structural similarity index measure (SSIM) were higher markedly than the values of the other two reconstruction methods. The diagnostic rate of routine MRI examination of the fallopian tube and other parts of the uterus was lower than or equal to that of MR-HSG examination by CNN. Routine MRI examinations of fallopian tube imaging artifacts were large, and the definition was reduced, which increased the difficulty of identification. However, MR-HSG examination by CNN indicated that the imaging artifacts were low, the clarity was high, and the influence of noise was small, which was conducive to clinical diagnosis and identification. For endometriosis, the accuracy of MR-HSG was 33.33% and the accuracy of MRI was 46.67%. CNN MR-HSG inspection method was significantly better than the conventional MRI inspection method (*P* < 0.05). Therefore, the results of this study revealed that MR-HSG examination by CNN had a clear imaging effect and obvious inhibition effect on background signals and rapid image generation without the need for reconstruction with the same spatial resolution, which improved the imaging quality and could provide a reference value for clinical diagnosis and subsequent related studies.

## 1. Introduction

The medical definition of female infertility is that no contraceptive measures have been taken for more than one year, and the sexual life is normal without a successful pregnancy. Besides, it is mainly divided into primary infertility and secondary infertility [1]. There are many causes of female infertility, mainly including ovulation disorders, fallopian tube factors, and abnormal endometrial receptivity. In clinical diagnostic examinations, imaging examinations are mainly relied on, combined with other examination methods, to improve the accuracy of diagnosis [2]. Moreover, fallopian tube obstruction is the most common anatomical cause of female infertility; imaging is the most common way to detect infertility in women, so the medical examination is used as the gold standard for evaluating the patency of the fallopian tube [3–5].

At present, the commonly applied clinical imaging examination methods mainly include hysterosalpingo-contrast-sonography (HSS) and HSG. The accuracy of these two methods is relatively similar, and the operation is simple and rapid, but only for patients with simple lesions. If accompanied by intrauterine fibroids endometriosis and other diseases, the status of the fallopian tube cannot be clearly evaluated [6].

MRI is the phenomenon of spin magnetic resonance, which has been discovered on the basis of solid-state microscopic quantum theory and the development of radio microwave electronics technology. Paramagnetic resonance was first observed in a water solution of paramagnetic Mn salt in 1945, and then, cyclotron resonance of electrons and holes was observed in semiconductor silicon and germanium. After these magnetic resonances have been discovered, they have been extensively used in basic disciplines such as physics, chemistry, biology, and new technologies such as microwave technology and quantum electronics [7]. Some advanced equipment manufacturers and researchers continue to optimize the performance of magnetic resonance scanners to further expand their advantages for better clinical application [8]. MRI has the advantages of simple operation and noninvasiveness. It is widely used in the field of obstetrics and gynecology and is a very mature technology. There are very rich adipose tissues around the female reproductive system. MRI can present multidirectional and high-resolution tissue imaging. Compared with inspection, it has obvious advantages. MRI can semiquantitatively study the endometrium, diffusion-weighted imaging, and reflect the changes around the endometrium through the movement of water molecules in the tissue. MRI has a good application prospect in the evaluation of the endometrium. MR-HSG is an examination method combining magnetic resonance with obstetrics and gynecology examination technology, which can analyze the causes of female infertility while evaluating the fallopian tube normality, and this technology has become a comprehensive method for evaluating female infertility [9].

Deep learning technology is to directly obtain data features from neural networks through the constructed deep-level network and perform adaptive extraction of features. It is widely used in medical image disease diagnosis. Some scholars apply the deep learning model to the computer. In the convolutional layer, define a convolution kernel, which can be regarded as a “receptive field.” When performing convolution operations, they are input in a certain order. After this processing, the characteristic of the input image can be obtained. As the depth of the network increases, the abstraction degree of the extracted image also increases, and the expressive ability becomes stronger and stronger. In order to improve the accuracy of the examination results, MRI image reconstruction technology based on CNN was applied in this study, which could add newly constructed network layers to the original convolutional network to achieve image reconstruction [10]. Therefore, 30 female patients with infertility were included in this study, all of which were examined by routine MRI and CNN-based MR-HSG to explore the diagnostic accuracy of the two examinations.

## 2. Materials and Methods

### 2.1. Research Objects

A total of 30 female patients with infertility, who were admitted to the hospital from May 2018 to January 2020, were selected as the research objects in this study. They were 23–35 years old, with an average age of 27.54 ± 5.24 years. The diagnostic criteria were referred to the relevant diagnostic criteria in Diagnostic Criteria and Interpretation of Infertility (Fertilization). The inclusion criteria were defined to include patients who met the above diagnostic criteria, had normal hormone levels and ovulation cycles, did not receive traditional HSG examinations, were not allergic to the relevant treatment and examination drugs used in the treatment or examination in this study, and were aware of and signed the informed consent forms (their family members also known and signed the informed consent forms). The exclusion criteria were defined to include patients who were combined with cognitive impairment, had poor compliance and were unable to cooperate with the researchers, recently received relevant medications, were accompanied with malignant tumors of the reproductive system, suffered from severe liver and kidney damage, and had contraindications to MRI examination. There is no significant difference compared with the general data of patients (*P* < 0.05).

### 2.2. Routine MRI Examination Method

Patients should receive routine MRI and MR-HSG examinations during the 7^th^–13^th^ day of the menstrual cycle. The patients should know the following instructions. Unprotected sex life should be avoided for two weeks before the examination to prevent pregnancy. Antibiotics should be taken or intravenously dropped before the examination, and oral sedatives could be taken to prevent nervousness and to avoid excessive artifacts during the procedure. Routine MRI examinations were performed using MRI diagnostic instrument (manufacturer: Siemens Avanto; model: Optima 360). According to testing parts, the body phase array surface coil was determined, and the desired scanning range was wrapped, so that it covered all the target area. The center of the front and rear coils should be in a straight line, and then, the belly strap should be fixed with moderate strength. The abdominal band should be tightened as far as possible to ensure that the patient felt comfortable without affecting the patient's breathing movement. The patient was asked to conduct breath-holding training, and the breath-holding time should be greater than 18 seconds. All sequences were performed with deep inhalation breath-hold scanning, including T1WI and T2WI sequences with transverse fast spin echo (FSE) sequences and axial FSE/T2WI sequences with short reversal time. The scanning layer spacing and thickness were set as 5.3 mm, and some patients could receive enhanced scanning after routine scanning. Accurate lesion scanning was performed according to the location of the lesion on the locator, and it took about 6–8 minutes to complete the routine scanning. The status of the patient was observed; if there was any abnormality, stop immediately and give appropriate treatment.

### 2.3. MR-HSG Examination Method

During the MR-HSG examination, it was necessary to prepare the contrast agent first. 25 mL of mixed contrast agent was adopted to add 2 mL of nuclear magnetic resonance contrast agent (Gd-DTPA) to 100 mL of iohexol. HSG applied 15 mL of iohexol so that the configuration was completed 30 minutes before operation and was stored in a 37°C incubator. 3–7 days after the end of the menstruation, each patient received the examination. Patients needed to take 20 mg of scopolamine butyrate orally 1 hour before surgery. After routine vulvar disinfection, a guiding speculum was introduced to expose the cervix. Then, the salpingography catheter was placed in the uterine cavity and injected with 2–3 mL of normal saline for fixation. These operations were performed by a radiologist with more than 5 years of routine HSG experience.

The German WideTEK 36 Art Scanner and 6-channel torso phased array surface coil were used for MRI image collection. Each patient was instructed to take a supine position and underwent a routine pelvic scanning. The scanning parameters were set as follows: in the axial SE T1WI sequence, repetition time (TR) was 709 ms, echo time (TE) was 16 ms, reversal angle was 138°, layer thickness was 3 mm, field of view (FOV) was 181 mm × 269 mm, and matrix was 224 × 320. In the axial SE T2WI sequence, TR was 5,799 ms, TE was 95 ms, reversal angle was 140°, layer thickness was 4 mm, FOV was 181 mm × 273 mm, and matrix was 256 × 320. In the sagittal SE T2WI sequence, TR was 3,020 ms, TE was 83 ms, reversal angle was 144°, layer thickness was 4 mm, FOV was 223 mm × 281 mm, and matrix was 255 × 321. The field of view of the dynamic enhanced scanning was positioned on this sequence, and 25 mL of contrast agent was manually injected, while the three-dimensional (3D) T1WI gradient echo dynamic enhanced scanning was performed simultaneously. Besides, TR was 3.24 ms, TE was 1.25 ms, reversal angle was 25°, layer thickness was 0.6 mm, FOV was 205 mm × 500 mm, and matrix was 360 × 384, with 4 periods of scanning (17 seconds of each period). Subtraction and maximum signal projection images were generated by reconstruction. Five minutes later, fast T1WI fat suppression sequence scanning (TR 3.78 ms, TE 1.41 ms, reverse angle 10°, layer thickness 4 mm, FOV 127 mm × 500 mm, and matrix 180 × 320) was performed to show pelvic contrast diffusion.

### 2.4. MRI Image Reconstruction Model Based on Convolutional Neural Network

Assuming that *x*€*A*^*n*^ was a 2D MR image complex signal spread into a column vector, *n* = *n*_*x*_*n*_*y*_, *y* = *A*_*m*_ (*m* < *n*) was the observation sample, *i*€*A*^*n*^ was the noise in the frequency-domain sampling process, and it was usually Gaussian noise. The imaging process of MR was as follows:

(1) $$\begin{array}{c} y=G_{u}x+i. \end{array}$$

Among them, *G*_*u*_€*A*^*m∗n*^ belonged to undersampling calculation, and *G*_*u*_ = *mG*. The equation included two steps. Firstly, the Fourier operator was employed to transform the MR signal into the domain, then the frequency-domain signal was for undersampling through the frequency-domain undersampling sample template *m*€*A*^*m∗n*^, and the index corresponding to the above sampling points was set as set *C*. The process of obtaining the original MRI signal *x* from the observation signal *y* was MRI image reconstruction.

The solution of equation (1) belonged to the reverse process of the image seeking problem. Since *G*_*u*_ was singular, the result obtained by direct inversion was not accurate. Therefore, in order to obtain stable and accurate results, it was necessary to use the prior knowledge of the image to solve, and the range of the solution can be restricted by adding a regular term, which could be expressed as follows:

(2) $$\begin{array}{c} xℑ\left(x\right)+\lambda {\left\|y-G_{u}x\right\|}_{2}^{2}. \end{array}$$

In equation (2), *ℑ*(*x*) stood for the regular term about *x* and *λ* represented the adjustment factor. The consistency of the reconstructed image and observed signal in the frequency domain was adjusted based on the noise level. In this process, CNN learned the abstract representation of the label sample (real image) and the abstract representation learned by the trained CNN could be used as the prior knowledge of the MR image and added to the optimization equation as a regular term as follows:

(3) $$\begin{array}{c} x\left\|x-g_{\text{cnn}}\left(\frac{x_{u}}{\theta }\right)\right\|Gu-y{\left\|\;\right.}_{2}^{2}. \end{array}$$

In equation (3), *θ* means the adjustable parameter, *g*_cnn_ stood for the forward propagation operation, the observation signal *y* was the aliased image obtained by the direct inverse Fourier transform, *x*_*u*_ was the input of CNN, and the reconstructed image was obtained through forward propagation.

In equation (3), *x*_*u*_ was obtained from undersampling, which violated the sampling theorem and caused aliasing in the time domain. Therefore, the reconstruction of CNN could be regarded as a time-domain antialiasing problem. The reconstructed image of the entire network could be expressed as follows:

(4) $$\begin{array}{c} x_{\text{cnn}}=g_{\text{cnn}}\left(\frac{x_{u}}{\theta },\lambda ,C\right). \end{array}$$

In order to promote the accuracy of reconstruction, this process was generally subdivided into two steps: reconstruction and data consistent operation.

During reconstruction, the training data *W* was input (*x*_*u*_, *x*_gnd_), *x*_gnd_ could represent the real image, and the network parameters were adjusted through the following equation to finally obtain the optimal network:

(5) $$\begin{array}{c} ℜ\left(\theta \right)=\sum_{x_{u},x_{\text{gnd}}}\lambda \left(x_{\text{gnd}},x_{\text{cnn}}\right). \end{array}$$

In equation (5), *λ* stood for the loss function, considering the mean square error loss:

(6) $$\begin{array}{c} \lambda \left(x_{\text{gnd}},x_{\text{cnn}}\right)={\left\|x_{\text{gnd}}-x_{\text{cnn}}\right\|}_{2}^{2}. \end{array}$$

Consistent data operation required the least square evaluation of equation (3):

(7) $$\begin{array}{c} \left(x-g_{\text{cnn}}\left(\frac{x_{u}}{\theta }\right)\right)+\lambda G_{u}^{L}\left(G_{u}x-y\right)=0. \end{array}$$

Equation (7) was simplified to get equation (8):

(8) $$\begin{array}{c} \left(P+\lambda G_{u}^{L}G_{u}\right)x=g_{\text{cnn}}\left(\frac{x_{u}}{\theta }\right)+\lambda G_{u}^{L}y. \end{array}$$

Fourier operator *G* was adopted to convert the data to frequency-domain calculation as follows:

(9) $$\begin{array}{c} \left(P+\lambda GG_{u}^{L}G_{u}G^{L}\right)Gx=Gg_{\text{cnn}}\left(\frac{x_{u}}{\theta }\right)+\lambda GG_{u}^{L}y, \end{array}$$

where *Y* = *Gx*_cnn_ = *Gg*_cnn_(*x*_*u*_*/θ*) and *T*=*GG*_*u*_^*l*^*y*, so equation (9) could be written as the main element operation form, which could be expressed as follows:

(10) $$\begin{array}{c} Y\left(i\right)=\left\{\begin{array}{cc} \frac{T_{\text{cnn}}\left(i\right)+\lambda T_{0}\left(i\right)}{1+\lambda }, & \text{if }i\in C,\\ T_{\text{cnn}}\left(i\right), & \text{if }i\notin C. \end{array}\right. \end{array}$$

During the image analysis, two physicians at the deputy director level would independently evaluate the patency of the fallopian tubes and other abnormalities. If the two sides disagreed, a third physician of the same level would participate in the evaluation.

### 2.5. MRI Image Structure Model Based on CNN

**Figure 1:**
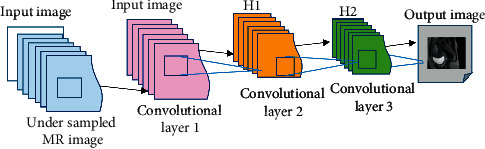
CNN-based MRI reconstruction model training structure diagram.

The convolutional neural network for MR image reconstruction mainly consists of an input layer, a convolutional layer, an activation layer, and an output layer. It contains three convolutional layers. The convolutional layer is composed of multiple feature surfaces, which are composed of multiple neurons. Each neuron is connected to the local area of the feature surface of the previous layer through the defined convolution kernel. The undersampled multichannel image block is used as the input of the network, and all fully sampled images are obtained through SOS (square root of the sum-of-squares) to acquire a single-channel fully sampled image, which is then used as the output label of the network (Figure 1).

### 2.6. Statistical Methods

Statistical analysis was performed using SPSS23.0 software. The Kolmogorov–Smirnov test was employed to test whether the measurement data conformed to the normal distribution. Normally distributed data were represented by $\left(\bar{x}\pm s\right)$, and those with skewed distribution were represented by the median (upper and lower quartiles) [*M*(*Q*1, *Q*3)]. The count data were represented by the number of cases and percentages. The Kappa value was applied to evaluate the consistency of MR-HSG and HSG in the diagnosis of fallopian tube patency. The Kappa value of 0.41–0.60 was moderately consistent, 0.61–0.80 was good, and 0.81–1.00 was good. The *χ*^2^ test was adopted to compare the differences between MRI and MR-HSG in the diagnosis of fallopian tubes. In addition, *P* < 0.05 indicated that the difference was statistically substantial.

## 3. Results

### 3.1. Evaluation of Reconstruction Results

The PSNR, NMSE, and SSIM of the inverse fast Fourier transform (IFFT), CNN_DC, and CNN with nondata conformance layer (CNN_NDC) were calculated, and the results are shown in Tables 1–3. It was found that the undersampling reconstruction results at 2, 4, and 6 times all showed that CNN_DC had a better effect, and its PSNR was lower steeply than the other two reconstruction methods. However, NMSE and SSIM were higher markedly than the index values of the other two reconstruction methods, and the differences were statistically huge (*P* < 0.05).

### 3.2. Comparison on the Two Examination Results

**Figure 2:**
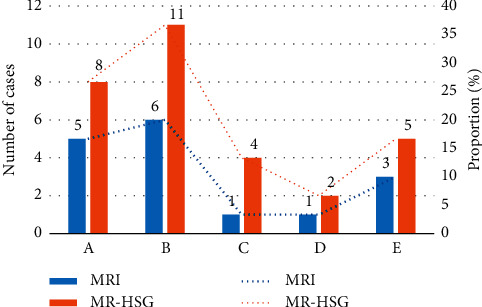
Comparison of the examination results of fallopian tubes (note: A: bilateral obstruction of fallopian tubes; B: bilateral partial obstruction; C: unilateral obstruction + unilateral partial obstruction; D: unilateral obstruction + unilateral partial obstruction; E: hydrosalpinx).

**Figure 3:**
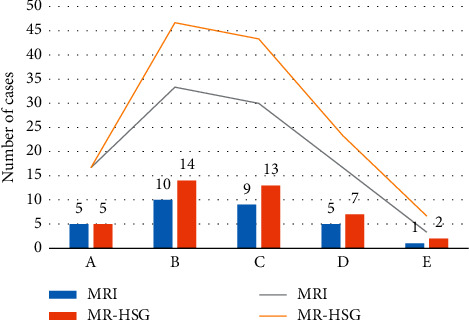
Comparison of the examination results of other lesions of the uterus (note: A: uterine adenomyosis; B: endometriosis; C: pelvic inflammatory disease; D: uterine fibroids; E: contrast countercurrent).

Figures 2 and 3 indicate that the diagnostic rate of routine MRI examination of fallopian tubes and other parts of the uterus was lower than or equal to the rate of MR-HSG examination by CNN, with a statistically marked difference (*P* < 0.05).

### 3.3. Comparison of the Two Imaging Examination Results

**Figure 4:**
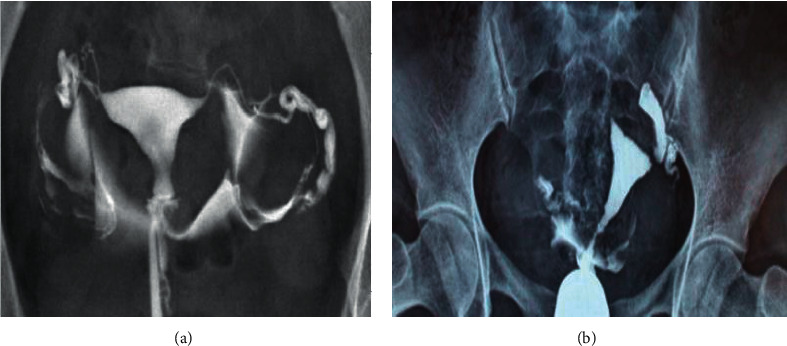
Results of routine MRI examination. (a) The obstructed left fallopian tube; (b) the poorly obstructed left fallopian tube.

**Figure 5:**
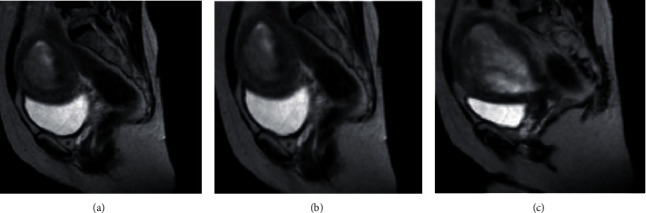
MR-HSG examination results. (a) Left fallopian tube obstruction; (b) bilateral fallopian tube obstruction; (c) proximal fallopian tube obstruction.

There is no significant difference compared with the general data of patients. The results of MRI and MR-HSG detection of fallopian tubes by randomly selected patients are shown in Figures 4 and 5. Figures 4 and 5 disclose that the routine MRI examination of the fallopian tube had large imaging artifacts, and the definition was reduced, which increased the difficulty of identification. What is more, the MR-HSG examination by CNN indicated that the imaging artifacts were low, the definition was high, and the impact of noise was small, which was conducive to clinical diagnosis.

## 4. Discussion

In recent years, fallopian tube lesions have gradually become the main cause of female infertility. Current clinical examination methods can clearly understand uterine-related lesions and fallopian tube morphology. However, routine imaging examinations have many shortcomings, including radiation, iodine-containing contrast agent, allergy, and infection, which have a great impact on patients [11–13]. MRI is a means of tomography examination and the use of static magnetic fields and protons in the body together to generate radio frequency pulses, thereby being secondary to the phenomenon of magnetic resonance. When the reaction stops, the MR signal can appear. The amount of information provided by MRI is far greater than that of many other imaging examinations. Therefore, it has great potential advantages for the diagnosis of diseases. It can directly make transverse, sagittal, coronal, and inclined plane images and will not produce artifacts similar to CT detection. The detection effect of the craniocerebral tumor, hematoma, hydrocephalus, and other common diseases is good, and it is also effective in diagnosing diseases such as lumbar posterior disc protrusion and primary liver cancer. This study showed that the accuracy rate of MR-HSG for endometriosis was 33.33%, the accuracy rate of MRI was 46.67%, the proportion of pelvic inflammatory cases detected by MR-HSG was 43.3%, and the proportion of cases detected by MRI was 43.3%. The ratio was 30%. The detection rate of MR-HSG for uterine fibroids was 23.33%, and the detection rate of MRI was 16.67%. The detection accuracy of MR-HSG was significantly higher than that of MRI. Compared with the routine examination of obstetrics and gynecology, MRI greatly reduces radiation damage [14] and can accurately display the internal organs and surrounding tissue structure of the pelvic cavity [15, 16], thus showing significant advantages in the differential diagnosis of some reproductive system diseases. MR-HSG combines MRI technology with the gynecological examination, which greatly reduces radiation damage and greatly improves the accuracy of detection results [17].

MR-HSG is an imaging examination method that uses multisequence and thick-layer projection, which generates images while scanning, which accurately records the scanning process, with the advantage of high spatial resolution [18, 19]. MR-HSG technology makes the fallopian tube visible, which is convenient for doctors' clinical differential diagnosis. At present, this examination method has become a research hotspot. The CNN in the deep learning algorithm was adopted in this study to reconstruct the MR-HSG imaging, which could markedly improve the imaging quality. The results of this study revealed that the undersampling reconstruction results at 2, 4, and 6 times all showed that CNN_DC had a better effect, and its PSNR was sharply lower than the other two reconstruction methods, while NMSE and SSIM were higher hugely than the other two reconstruction methods. Thus, it showed that the reconstruction effect of CNN_DC based on CNN was good, which was conducive to improving the clarity of imaging, reducing artifacts, and decreasing other interference.

Volondat et al. [20] compared and analyzed the diagnostic accuracy of MRI hysterosalpingography and conventional hysterosalpingography for female infertility. The scores for the detection of the fallopian tube or intraluminal abnormalities between patients and scorers were 0.92 and 0.76 uterus. Salpingogram can be used for a single comprehensive examination of female infertility. In this study, the currently applied contrast agent was adopted, and the contrast agent injection method was emphasized. During the examination, it was found that the intrauterine environment and the fallopian tube under the dynamic scanning were clearly visible, and the continuity was good. This technology had an obvious inhibition effect on background signal, relatively rapid image generation, and no need for reconstruction. The imaging results suggested that the imaging artifacts of routine MRI examination of the fallopian tube were larger, and the definition was reduced, which rose the difficulty of identification. The MR-HSG examination by CNN found that the imaging artifacts were low, the definition was high, and the noise influence was small, which was conducive to clinical diagnosis and discrimination, thereby confirming the superiority of the MR-HSG examination. Through the analysis of the imaging results, it was found that the diagnosis rate of routine MRI examination of the fallopian tube and other parts of the uterus was lower than or equal to the diagnosis rate of the MR-HSG examination by CNN. Therefore, the MR-HSG examination by CNN was applicable for the clinical diagnosis of infertility.

## 5. Conclusion

This study used the convolution neural network-based MRI image reconstruction technology, adding a new network layer to the original convolution network to achieve image reconstruction. 30 cases of female infertility patients were selected, all underwent conventional magnetic resonance, and convolution neural network-based magnetic resonance salpingography was undertaken to explore the diagnostic accuracy of the two methods. The results showed that the imaging effect of MR-HSG examination by CNN was clear, the background signal suppression effect was obvious, the image was generated quickly, no reconstruction was required, the spatial resolution was the same, the imaging quality was improved, and it was worthy of further clinical promotion and use.

The disadvantage of this study is that it fails to record the imaging results completely, which results in the loss of part of the data and could not fully reflect the entire research process.

The sample size in the research is too small, and it needs to be expanded to illustrate the accuracy of the research results. Therefore, further research is needed to improve the clinical reference value.

## Figures and Tables

**Table 1:** Evaluation of MRI image reconstruction results based on CNN (2-time undersampling reconstruction).

Reconstruction methods/indicators	PSNR	NMSE	SSIM
IFFT	0.132	30.69	0.785
CNN_DC	0.015	48.76	0.996
CNN_NDC	0.059	37.72	0.971

**Table 2:** Evaluation of MRI image reconstruction results based on CNN (4-time undersampling reconstruction).

Reconstruction methods/indicators	PSNR	NMSE	SSIM
IFFT	0.310	23.62	0.668
CNN_DC	0.056	38.89	0.943
CNN_NDC	0.137	30.16	0.901

**Table 3:** Evaluation of MRI image reconstruction results based on CNN (6-time undersampling reconstruction).

Reconstruction methods/indicators	PSNR	NMSE	SSIM
IFFT	0.276	24.55	0.672
CNN_DC	0.068	36.31	0.941
CNN_NDC	0.147	29.92	0.892

## Data Availability

The data used to support the findings of this study are available from the corresponding author upon request.

## References

[1] Liang N., Wu Q.-Q., Li J.-H., Gao F.-Y., Sun F.-L., Guo C.-X. (2019). Causes of misdiagnosis in assessing tubal patency by transvaginal real-time three-dimensional hysterosalpingo-contrast sonography. *Revista da Associação Médica Brasileira*.

[2] Gao Y. B., Yan J. H., Yang Y. D., Sun J., Dong J. Y., Cui G. H. (2019). Diagnostic value of transvaginal four-dimensional hysterosalpingo-contrast sonography combined with recanalization in patients with tubal infertility. *Nigerian Journal of Clinical Practice*.

[3] Promberger R., Simek I.-M., Nouri K., Obermaier K., Kurz C., Ott J. (2018). Accuracy of tubal patency assessment in diagnostic hysteroscopy compared with laparoscopy in infertile women: a retrospective cohort study. *Journal of Minimally Invasive Gynecology*.

[4] Chen S., Du X., Chen Q., Chen S. (2019). Combined real-time three-dimensional hysterosalpingo-contrast sonography with B mode hysterosalpingo-contrast sonography in the evaluation of fallopian tube patency in patients undergoing infertility investigations. *BioMed Research International*.

[5] Groszmann Y. S., Benacerraf B. R. (2016). Complete evaluation of anatomy and morphology of the infertile patient in a single visit; the modern infertility pelvic ultrasound examination. *Fertility and Sterility*.

[6] Kemfang J. D., Kasia J. M., Georges N.-T., Nkongo V., Sone C., Fongang E. (2015). Comparison of hysterosalpingo grams with laparoscopy in the diagnostic of tubal factor of female infertility at the Yaoundé general hospital, Cameroon. *Pan African Medical Journal*.

[7] Wang R., van Welie N., van Rijswijk J. (2019). Effectiveness on fertility outcome of tubal flushing with different contrast media: systematic review and network meta‐analysis. *Ultrasound in Obstetrics and Gynecology*.

[8] Malik B., Patil S., Boricha B. G., Kurkal N., Choudhry M. (2014). A comparative study of the efficacy of sonosalpingography and hysterosalpingogram to test the tubal patency in all women with primary and secondary infertility. *Ultrasound Quarterly*.

[9] Alcázar J. L., Martinez-Astorquiza Corral T., Orozco R., Dominguez-Piriz J., Juez L., Errasti T. (2016). Three-dimensional hysterosalpingo-contrast-sonography for the assessment of tubal patency in women with infertility: a systematic review with meta-analysis. *Gynecologic and Obstetric Investigation*.

[10] Robertshaw I. M., Sroga J. M., Batcheller A. E. (2016). Hysterosalpingo-contrast sonography with a saline-air device is equivalent to hysterosalpingography only in the presence of tubal patency. *Journal of Ultrasound in Medicine*.

[11] Calles-Sastre L., Engels-Calvo V., Ríos-Vallejo M. (2018). Prospective study of concordance between hysterosalpingo-contrast sonography and hysteroscopy for evaluation of the uterine cavity in patients undergoing infertility studies. *Journal of Ultrasound in Medicine*.

[12] Ahinko-Hakamaa K., Huhtala H., Tinkanen H. (2007). The validity of air and saline hysterosalpingo-contrast sonography in tubal patency investigation before insemination treatment. *European Journal of Obstetrics & Gynecology and Reproductive Biology*.

[13] Hajishafiha M., Zobairi T., Zanjani V. R., Ghasemi-Rad M., Yekta Z., Mladkova N. (2009). Diagnostic value of sonohysterography in the determination of fallopian tube patency as an initial step of routine infertility assessment. *Journal of Ultrasound in Medicine*.

[14] Lim S. L., Jung J. J., Yu S. L., Rajesh H. (2015). A comparison of hysterosalpingo-foam sonography (HyFoSy) and hysterosalpingo-contrast sonography with saline medium (HyCoSy) in the assessment of tubal patency. *European Journal of Obstetrics & Gynecology and Reproductive Biology*.

[15] Habibaj J., Kosova H., Bilali S., Bilali V., Qama D. (2012). Comparison between transvaginal sonography after diagnostic hysteroscopy and laparoscopic chromopertubation for the assessment of tubal patency in infertile women. *Journal of Clinical Ultrasound*.

[16] Heikkinen H., Tekay A., Volpi E., Martikainen H., Jouppila P. (1995). Transvaginal salpingosonography for the assessment of tubal patency in infertile women: methodological and clinical experiences. *Fertility and Sterility*.

[17] Yuan L., Jingying H., Xiujuan C. (2019). Predictive value of a modified classification of fallopian tube status on prognosis of tubal factor infertility after laparoscopic surgery. *Medicine (Baltimore)*.

[18] Exacoustos C., Zupi E., Szabolcs B. (2009). Contrast-tuned imaging and second-generation contrast agent SonoVue: a new ultrasound approach to evaluation of tubal patency. *Journal of Minimally Invasive Gynecology*.

[19] Farhi J., Weissman A., Nahum H., Levran D. (2000). Zygote intrafallopian transfer in patients with tubal factor infertility after repeated failure of implantation with in vitro fertilization-embryo transfer. *Fertility and Sterility*.

[20] Volondat M., Fontas E., Delotte J., Fatfouta I., Chevallier P., Chassang M. (2019). Magnetic resonance hysterosalpingography in diagnostic work-up of female infertility - comparison with conventional hysterosalpingography: a randomised study. *European Radiology*.

